# Identification of *Eimeria* spp. in domestic chickens raised in alternative poultry production systems in the State of São Paulo, Brazil

**DOI:** 10.1590/S1984-29612023075

**Published:** 2023-12-04

**Authors:** José Carlos Soares, Bruno Ferraz Itoyama, Bruna Matarucco Sampaio Beretta, Camila Michele de Souza Hossotani, Maria Santa Cardoso Silva, Giane Serafim da Silva, Alex Akira Nakamura, Flávia Lombardi Lopes, Marcelo Vasconcelos Meireles

**Affiliations:** 1 Faculdade de Medicina Veterinária, Universidade Estadual Paulista – UNESP, Araçatuba, SP, Brasil; 2 Instituto Biológico, Agência Paulista de Tecnologia Agropecuária, Votuporanga, SP, Brasil

**Keywords:** Coccidiosis, poultry, molecular diagnosis, next generation sequencing, Coccidiose, aves, diagnóstico molecular, sequenciamento de nova geração

## Abstract

The objective of this study was to identify *Eimeria* spp. in alternative poultry production systems (APPS) in the State of São Paulo, Brazil. Fecal samples (168) and DNA extracted from fecal samples obtained in APPS located in different Municipalities in the State of São Paulo (93) were examined by microscopy or genera-specific PCR (ITS-1 *locus*). Samples positive for *Eimeria* spp. were examined using *Eimeria lata*, *Eimeria nagambie,* and *Eimeria zaria* species-specific PCR protocols (ITS-2 *locus*) and another *E. lata*-specific PCR (candidate IMP1 genomic *locus*) followed by molecular cloning (*E. lata* and *E. zaria* ITS-2 amplicons) and genetic sequencing. All positive DNA samples were also submitted to genera-specific nested PCR (18S rRNA gene) followed by next-generation sequencing to identify *Eimeria* spp. *Eimeria nagambie*, *E. zaria*, and *Eimeria* sp. were identified by ITS2-targeted species-specific PCRs and genetic sequencing. Next-generation sequencing identified, in order of prevalence: *E. nagambie*; *Eimeria acervulina*; *Eimeria mivati*; *Eimeria praecox*; *Eimeria brunetti*; *Eimeria mitis*; *Eimeria* sp.; *Eimeria maxima*; *E. zaria,* and *Eimeria necatrix*/*tenella*. Our results confirmed, for the first time in Brazil, the identification of *E. nagambie*, *E. zaria*, and *Eimeria* spp. ITS-2 and 18S rRNA gene sequences not yet described in Brazil.

## Resumo

O objetivo deste trabalho foi identificar *Eimeria* spp. em galinhas domésticas criadas em sistemas de criação alternativos (SCA). Foram utilizadas 93 amostras de DNA e 168 amostras de fezes de galinhas provenientes de SCA, localizados em 17 municípios do estado de São Paulo. As amostras foram examinadas por microscopia ou PCR gênero-específica (*locus* ITS-1); aquelas positivas foram examinadas por PCRs espécie-específicas para *Eimeria lata*, *Eimeria nagambie* e *Eimeria zaria* (*locus* ITS-2), seguidas de clonagem (*E. lata* e *E. zaria*) e sequenciamento genético, e por outro protocolo de PCR específico para *E. lata* (*locus* IMP1). Adicionalmente, as mesmas amostras foram submetidas à *nested* PCR gênero-específica (gene 18S rRNA), seguida de sequenciamento de nova geração para identificação de *Eimeria* spp. *Eimeria nagambie*, *E. zaria* e *Eimeria* sp. foram identificadas pela PCR espécie-específica e sequenciamento genético. O sequenciamento de nova geração identificou, em ordem de prevalência: *E. nagambie, Eimeria acervulina, Eimeria mivati, Eimeria praecox, Eimeria brunetti, Eimeria mitis, Eimeria* sp., *Eimeria maxima, E. zaria* e *Eimeria necatrix/tenella*. Os resultados observados confirmaram, pela primeira vez no Brasil, a identificação de *E. nagambie*, *E. zaria* e de sequências de *Eimeria* spp. ainda não descritas no Brasil, referentes aos genes ITS-2 e 18S rRNA.

## Introduction

Coccidiosis is one of the most economically relevant diseases for the poultry industry (Williams, 1999; Blake et al., 2020). Seven species of *Eimeria* infect the domestic chicken: *Eimeria acervulina*, *Eimeria brunetti*, *Eimeria mitis*, *Eimeria maxima*, *Eimeria necatrix*, *Eimeria praecox*, and *Eimeria tenella* (Vrba et al., 2011).

Several studies have detected the occurrence of genetic variants in *Eimeria* populations in different countries (Morris et al., 2007; Cantacessi et al., 2008; Fornace et al., 2013; Jatau et al., 2016). Three genetic variants, which Cantacessi et al. (2008) called operational taxonomic units (OTUs) x, y, and z were proposed as novel species of domestic chickens: *Eimeria lata*, *Eimeria nagambie*, and *Eimeria zaria*, respectively (Blake et al., 2021). The nomenclature proposed by Blake et al. (2021) is adopted throughout this manuscript, especially when studying cryptic species (Allgayer et al., 2021).

The occurrence of *E. lata, E. nagambie*, and *E. zaria* has been reported in alternative and industrial poultry farming systems in several countries (Jatau et al., 2016; Fornace et al., 2013; Hinsu et al. 2018; Godwin & Morgan, 2015; Morgan & Godwin, 2017). However, there is a lack of information about the pathogenesis and epidemiology of these new species. This is of great concern when it comes to their prevalence and epidemiological relevance, including the effectiveness of current control measures against coccidiosis, particularly of vaccination against eimeriosis (Venkatas & Adeleke, 2019), since current vaccines against eimeriosis do not contain these three species, which can infect chickens previously vaccinated (Godwin & Morgan, 2015; Blake et al., 2021).

*Eimeria lata, E. nagambie*, and *E. zaria* infect from the middle part of the duodenum to the distal part of the ileum (Cantacessi et al., 2008; Blake et al., 2021) and adversely affect production parameters in broilers and laying hens (Fornace et al., 2013). Moreover, mortality in broiler chickens has been attributed to *E. lata* and *E. nagambie* (Morris et al., 2007). Depending on the number of inoculated oocysts, the reduction in weight gain can reach 28.8% and 31.1% in *E. lata* and *E. nagambie* infections, respectively (Blake et al., 2021).

Considering the relevance of coccidiosis for the health of poultry and for Brazil’s economy, data on the prevalence of *Eimeria* spp. in commercial poultry production systems (CPPS) are outdated and scanty in alternative poultry production systems (APPS). Information has been reported about the microscopic or molecular identification of *E. acervulina*, *E. brunetti*, *E. maxima*, *E. mitis/mivati*, *E. necatrix*, *E. praecox*, and *E. tenella* in CPPS in Brazil (Terra et al., 2001; Santos et al; 2003; Meireles et al., 2004; Luchese et al., 2007; Carvalho et al., 2011; Moraes et al., 2015). However, although *E. acervulina*, *E. brunetti*, *E. maxima*, *E. mitis/E. mivati*, *E. necatrix*, *E. praecox*, and *E. tenella* have been identified based on morphological and morphometric data (Luchese et al., 2007; Noronha et al., 2020; Silva et al., 2022), no studies so far have focused on the molecular identification of *Eimeria* spp. in APPS in Brazil. Therefore, the objective of this study was to investigate the occurrence of infection by *Eimeria* spp. and potential new OTUs in APPS in the State of São Paulo, Brazil.

## Material and Methods

### Fecal and DNA samples

**Figure 1:**
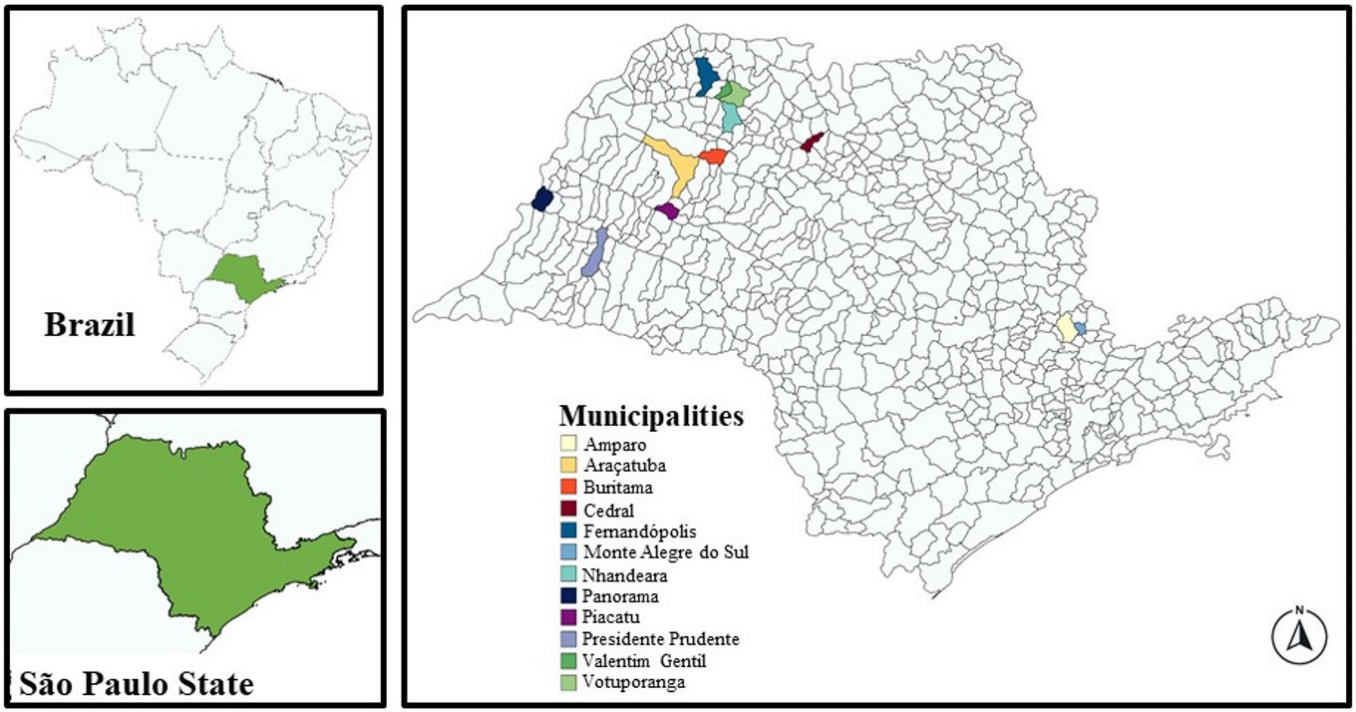
Location of Municipalities of the State of São Paulo, Brazil, where chicken feces samples were collected from alternative poultry production systems.

Fecal and DNA samples originated from asymptomatic chickens raised in APPS in the State of São Paulo (Figure 1). APPS consisted of extensive and semi-intensive broiler and layer production systems located in rural areas, each containing six to 250 chickens of several ages and breeds. Chickens have never been vaccinated against coccidiosis and were not medicated in the weeks prior to sample collection. A total of 261 samples were evaluated: 93 samples consisted of genomic DNA samples stored at -20ºC for approximately four years, which were used in a previous study related to *Cryptosporidium* spp. (Santana et al., 2018). These samples were extracted from the feces of domestic chickens that were collected by convenience sampling from APPS located in 12 municipalities. The remaining 168 samples consisted of feces collected by convenience sampling from APPS located in seven municipalities, in 2021. Each fecal and DNA sample originated from one pool of recently eliminated feces, from up to 10 chickens per APPS, which were picked up with a disposable wooden spatula and preserved in 2.5% potassium dichromate at 4°C.

### Screening for *Eimeria* spp. by genus-specific PCR and microscopy

Screening for *Eimeria* spp. in DNA samples extracted in 2018 was performed using a genus-specific PCR targeting the internal transcribed spacer (ITS)-1 locus (Lew et al., 2003) (Table 1) and Jumpstart™ Taq ReadyMix (Sigma Aldrich), in a SimpliAmp® thermal cycler (Thermo Fisher Scientific). The screening was performed in the following conditions: initial DNA denaturation at 94°C for 2 minutes, followed by 35 cycles, each consisting of denaturation at 94°C for 30 seconds, annealing at 55°C for 30 seconds, and extension at 72°C for 1 minute, with a final extension cycle at 72°C for 7 minutes. Genomic DNA extracted from *Eimeria* oocysts from the vaccine Bio-Coccivet R (Vaxxinova Biovet Brazil) was used as a positive control. Ultrapure water was used as a negative control.

**Table 1:** Molecular protocols used to detect and identify *Eimeria* spp. in alternative poultry production systems.

**Protocol**	**Primer**	**Primer sequence**	**Amplicon size (bp)**	***Eimeria* Species / OTU**	**Target gene**	**Reference**
PCR	EF1	AAGTTGCGTAAATAGAGCCCTC	317-585	*Eimeria* spp.	ITS-1	Lew et al. (2003)
	ER1	AGACATCCATTGCTGAAAG				
PCR/cloning/sequencing	OTUXfor	GTGGTGTCGTCTGCGCGT	134	*E. lata*	ITS-2	Fornace et al. (2013)
	OTUXrev	ACCACCGTATCTCTTTCGTGA				
PCR/cloning/sequencing	OTUZfor	TATAGTTTCTTTTGCGCGTTGC	154	*E. zaria*		
	OTUZrev	CATATCTCTTTCATGAACGAAAGG				
PCR/sequencing	OTUYfor	CAAGAAGTACACTACCACAGCATG	347	*E. nagambie*		
	OTUYrev	ACTGATTTCAGGTCTAAAACGAAT				
PCR	OTU-Xf2	GGGTAGAGCCAGGGGTAGAG	1,018	*E. lata*	IMP1 genomic locus	Blake et al. (2021)
	OTU-Xr2	CGTAGTCCCAAGTGCCAACT				
Nested PCR/NGS	18S-F-out	CGGGTAACGGGGAATTAGGG	538	*Eimeria* spp.	18S rRNA	Hauck et al. (2019)
	18S-R-out	TACGAATGCCCCCAACTGTC				
	18S-F-in*	TCGTCGGCAGCGTCAGATGTGTATAAGAGACAGATTGGAGGGCAAGTCTGGTG	260			
	18S-R-in^*^	GTCTCGTGGGCTCGGAGATGTGTATAAGAGACAGTGCTGCAGTATTCAGGGCRA				

* Sequences of the Illumina adapters are underlined.

Fecal samples collected in 2021 were screened for oocysts of *Eimeria* spp. by microscopy using a simple salt flotation technique. Oocysts from positive samples were purified by centrifugal flotation in a sucrose solution. DNA samples were extracted from the pellet from the purification protocol using a GenElute™ Stool DNA Isolation Kit (Sigma Aldrich) and were stored at -20°C.

### Screening for *E. lata, E. nagambie*, and *E. zaria* by species-specific PCR

All the samples positive by genus-specific PCR or by microscopy were subjected to species-specific PCR protocols (Table 1) targeting the ITS-2 locus of *E. lata* (134 pb), *E. nagambie* (347 bp), and *E. zaria* (154 bp) (Fornace et al., 2013). Each reaction consisted of a total volume of 25 μl containing 12.5 μl of JumpStart Taq ReadyMix (Sigma Aldrich), 2.5 μl of target DNA, 200 nM of each primer, and ultrapure water, under the following conditions: initial DNA denaturation at 94° C for 2 min, followed by 39 cycles, each cycle consisting of denaturation at 94°C for 30 s, annealing at 60°C for 30 s, extension at 72°C for 30 s, and final extension at 72°C for 7 min. Positive controls for *E. lata*, *E. nagambie*, and *E. zaria* PCRs consisted of DNA samples that were previously diagnosed as positive by species-specific PCRs (Fornace et al., 2013). Ultrapure water was used as a negative control.

Additionally, samples positive by PCR specific for *E. lata* (Fornace et al., 2013) were further examined using another PCR protocol specific for *E. lata* (1018 bp) with the primers OTU-Xf2 and OTU-Xr2 (Blake et al., 2021) (Table 1), under the following conditions: initial DNA denaturation at 94°C for 2 min, followed by 40 cycles, each cycle consisting of denaturation at 94°C for 30 s, annealing at 58°C for 30s, extension at 72°C for 60 s, and a final extension cycle at 72°C for 7 min. Plasmids containing the synthetic PCR targeted DNA sequence (candidate IMP1 genomic locus) (GenScript) of *E. lata* and ultrapure water were used as positive and negative controls, respectively.

Amplicons from PCRs targeting the ITS-2 sequences of *E. lata* and *E. zaria* were purified using a QIAquick™ Gel Extraction Kit (Qiagen) and then cloned using a TransformAid™ Bacterial Transformation Kit (Thermo Fisher Scientific) and a CloneJET™ PCR Cloning Kit (Thermo Fisher Scientific).

PCR amplicons from *E. nagambie* and plasmids from *E. lata* and *E. zaria* specific PCRs were purified using ExoSAP-IT™ PCR Product Cleanup Reagent (Thermo Fisher Scientific) and a GenElute™ HP Five-Minute Plasmid Miniprep Kit (Sigma-Aldrich), respectively. They were then sequenced in both directions using the ABI Prism™ Dye Terminator 3.1, on an ABI 3730XL automatic sequencer (Applied Biosystems), at the Sequencing and Functional Genomics Center of UNESP *Campus* Jaboticabal, SP, Brazil. Sequences were analyzed using CodonCode Aligner version 9.0.1 (CodonCode Corporation), BioEdit Sequence Alignment Editor (Hall, 1999), and the Basic Local Alignment Search Tool (BLAST).

### Nested PCR and next-generation genetic sequencing to detect and identify *Eimeria* spp.

Samples positive for *Eimeria* spp. by microscopy or PCR plus four negative samples were further examined by genera-specific nested PCR targeting the 18S rRNA gene of *Eimeria* spp., followed by next-generation sequencing (NGS) (Hauck et al., 2019) to detect and identify *Eimeria* spp. and potential new OTUs. Overhang adapters compatible with Illumina MiSeq index and sequencing adapters were added to the 5' end of nested PCR primers (Illumina, 2013) (Table 1).

PCR protocols were performed using Jumpstart™ Taq ReadyMix (Sigma Aldrich), under the following conditions. Preparation of 25μL of a solution containing 12.5 μL of Jumpstart™ Taq ReadyMix (Sigma Aldrich), 400 nM (PCR) or 800 nM (nested PCR) of each primer, and 2.5 μL (PCR) or 1 μL (nested PCR) of target DNA. Samples were subjected to initial denaturation for 2 min at 94º C followed by 35 cycles of denaturation at 94º C for 30 s, annealing at 60º C for 30 s, and extension at 72º C for 30 s, followed by a final cycle at 72º C for 7 min, using a SimpliAmp™ thermal cycler (Thermo Fisher Scientific). Genomic DNA extracted from oocysts from the commercial vaccine Bio-Coccivet R (Biorad) and ultrapure water were used as positive and negative controls.

Nested PCR amplicons were visualized by agarose gel electrophoresis, purified with a ProNex™ Size-Selective Purification System (Promega), and quantified using a Qubit™ digital fluorimeter (Thermo Fisher Scientific).

Samples were processed according to the Illumina 16S metagenomic protocol (Illumina, 2013), with 150 bp paired-end reads, using MiSeq™ Reagent kit v2 (Illumina). Libraries were prepared using 1 µl of the nested PCR amplicon, regardless of the quantification result. Amplification reactions were performed in a volume of 50 µl containing 5 µl of Nextera XT™ index primer 1 (N7xx), 5 µl of Nextera XT™ index primer 2 (S5xx), 25 µl of Kapa™ Hot Start High Fidelity Ready Mix (Kapa Biosystems), and 14 µl of ultrapure water. Samples were denatured at 95º C for 3 min, followed by 8 cycles of denaturation at 95º C for 30 s, annealing at 55º C for 30 s, and extension at 72º C for 30 s, with a final extension cycle at 72°C for 5 min.

Libraries were purified with a ProNex™ Size-Selective Purification System (Promega), quantified using a Qubit™ digital fluorimeter (Thermo Fisher Scientific), and normalized to a final DNA concentration of 8 pM. PhiX control library was spiked at a concentration of 15%.

Library sequencing was carried out at the Laboratory of Epigenomics of the Faculdade de Medicina Veterinária, UNESP *Campus* Araçatuba in a MiSeq™ sequencer (Illumina). Adapter sequences were trimmed according to Illumina FASTQ file generation pipelines included in the Illumina Experimental Manager software. Sequences were analyzed using MetaAmp Version 3.0 - OTU based amplicon analysis (Dong et al., 2017). Further analyses to detect chimeras were performed in OTUs originating from MetaAmp analyses using the chimera.uchime algorithm (Edgar et al., 2011) available on the Galaxy platform (The Galaxy Community, 2022).

A given species/OTU was considered to be present in each sample provided that its sequences: 1) corresponded to more than 1% of the sample sequences; 2) were grouped in the same cluster; and 3) were 97% or more genetically similar to the reference sequences. Representative sequences from each species/OTU were compared with sequences from *Eimeria* spp. using BLAST searches.

Nucleotide sequences generated in this study were submitted to the GenBank database under accession numbers OR229147-OR229154 and OR226404-OR226414 (Tables 2 and 3).

**Table 2:** Sequences obtained by *E. lata*, *E. nagambie*, and *E. zaria* ITS-2 gene-targeted specific PCR (Fornace et al., 2013), cloning, and sequencing of fecal samples from domestic chickens raised in alternative poultry production systems.

**Species-specific PCR (No. positive/ No. sampled; % positive)**	**Identification by cloning** ^*^ **and genetic sequencing (No. samples)**	**GenBank accession numbers from this study**	**Genetic similarity to sequences published in the GenBank database**		
			**Species**	**Accession numbers**	**%**
					
*E. lata* (6/80; 7.5)	*Eimeria* sp. (1)	OR229147	*E. lata*	HE997168	99.3
	*Eimeria* sp. (2)	OR229148	*E. maxima*	FJ230377	98.5
	*Eimeria* sp. (1)	OR229149	*E. lata*	AM922252	97.8
	*Eimeria* sp. (2)	OR229150	*Eimeria* sp.	LN609922	97.8
*E. nagambie* (15/80; 18.8)	*E. nagambie* (1)	OR229151	*E. nagambie*	AM922253	100
	*Eimeria* sp. (1)	OR229152	*Eimeria* sp.	AM922253	98.3
*E. zaria* (17/80; 24)	*E. zaria* (1)	OR229153	*E. zaria*	HE997165	100
	*E. zaria* (1)	OR229154	*E. zaria*	LT549041	100

* Cloning was performed only in amplicons from *E. lata* (2) and *E. zaria* (2).

**Table 3:** Identification of *Eimeria* spp. in fecal samples from chickens raised in alternative poultry production systems by nested PCR targeting the 18S rRNA gene and next-generation sequencing (Hauck et al., 2019).

***Eimeria* species**	**GenBank accession numbers from this study**	**% similarity to GenBank sequences**	**No. positive/ No. sampled (% positive)**	**Number of sequences**
*E. nagambie*	OR226404	100 (LT964973)	41/84 (48.8)	94,160
*E. acervulina*	OR226405	100 (KT184333)	39/84 (46.4)	133,330
*E. mivati*	OR226406	100 (FJ236377)	37/84 (44)	59,724
*E. praecox*	OR226407	100 (KT184352)	36/84 (42.9)	147,810
*E. brunetti*	OR226408	100 (EBU67116)	33/84 (39.3)	73,285
*E. mitis*	OR226409	99.6 (FR775303)	32/84 (38.1)	32,102
*Eimeria* sp.	OR226410	100 (MN073208)	29/84 (34.5)	53,189
*E. maxima*	OR226411	99.1 (FJ236335)	20/84 (23.8)	57,044
*E. maxima*	OR226412	100 (FJ236357)	15/84 (17.9)	32,951
*E. maxima*	OR226413	99.6 (FJ236361)	12/84 (14.3)	9,949
*E. zaria*	OR226414	99.6 (LT964974)	21/84 (25)	34,005
*E. necatrix/tenella* ^*^	-	100 (DQ136177)	20/84 (23.8)	42,227
		100 (DQ136185)		
*E. lata*	-	-	0/84 (0%)	0

* Sequencing could not distinguish between *E. necatrix* and *E. tenella*. The sequence was not uploaded to the GenBank database.

## Results

Using genus-specific PCR and microscopy, 33.3% (31/93) and 29.2% (49/168) samples positive for *Eimeria* spp., respectively, were identified. All the samples positive for *Eimeria* spp. by genus-specific PCR or microscopy (80/261; 30.7%) were analyzed by species-specific PCRs (see results in Table 2).

All the samples positive for *E. lata* (6/80; 7.5%) by the protocol of Fornace et al. (2013) were negative by the *E. lata*-specific PCR protocol of Blake et al. (2021). Four distinct sequences that showed greater genetic similarity to *E. lata*, *E. maxima*, or several sequences of *Eimeria* sp. were identified by genetic sequencing of ITS-2 plasmids from *E. lata*-specific PCR. These samples were thus classified as *Eimeria* sp. Two *E. zaria*-specific PCR sequences showed 100% genetic similarity with *E. zaria* sequences published in GenBank. Amplicons from *E. nagambie-*specific PCR showed two distinct genetic sequences: one sequence with 98.3% genetic similarity to *E. nagambie* was classified as *Eimeria* sp.; the other sequence had 100% genetic similarity with *E. nagambie* (Table 2).

Table 3 describes *Eimeria* species and the number of sequences obtained by the 18S rRNA gene next-generation sequencing. Although *E. mivati* 18S rRNA gene is currently considered a different type within *E. mitis* genome (Vrba et al., 2011), the sequences from our study were identified according to the species recorded in the GenBank database. *Eimeria necatrix* and *E. tenella* could not be differentiated by NGS of nested PCR amplicons.

The following species were identified, in order of prevalence: *E. nagambie*, *E*. *acervulina*, *E. mivati*, *E. praecox*, *E. brunetti*, *E. mitis*, unclassified *Eimeria* sp., *E. maxima*, *E. zaria*, and *E. necatrix/tenella*.

Mono-infections with *E. acervulina*, *E. maxima*, *E. mitis/mivati*, E. *nagambie*, *E. praecox*, *E. necatrix*/*tenella*, *E. zaria*, and unidentified *Eimeria* sp. were detected in 16/84 (19%) APPS. Mixed infections with *Eimeria* spp., including *E. necatrix/tenella* and unidentified *Eimeria* sp. were detected as follows: two species (11/84; 13.1%); three species (10/84 (11.1%); four species (8/84; 9.5%); five species (11/84; 13.1%); six species (11/84; 13.1%); seven species (4/84; 4.8%); and nine species (1/84; 1.2%).

All the samples were negative for *E. lata* by NGS. Sequences from unclassified *Eimeria* sp. identified in 34.5% of the samples exhibited 100% genetic similarity to *Eimeria* sp. 2 RHa-2020 (MN073208) described by Clark et al. (2016) in commercial broiler chickens in the United States (Table 3). *Eimeria acervulina*, *E. brunetti*, *E. maxima*, *E. mitis/mivati*, *E. necatrix*/*tenella*, and *E. praecox* were detected in vaccine Bio-Coccivet R.

In addition, NGS revealed a low prevalence of sequences representing *Eimeria* and *Isospora* species from other hosts, most closely related to *Eimeria bovis*, *Eimeria crandalis*, *Eimeria dispersa*, *Eimeria ferrisi, Eimeria inocua*, *Eimeria mandali*, *Eimeria mayurai*, *Eimeria meleagrimitis, Eimeria riyadhae,* and *Isospora* sp. ex *Myodes glareolus*.

## Discussion

The detection of seven species of *Eimeria* using molecular techniques has been reported in CPPS in Brazil, namely, *E. acervulina*, *E. brunetti*, *E. maxima*, *E. mitis*, *E. necatrix*, *E. praecox*, and *E. tenella* (Meireles et al., 2004; Carvalho et al., 2011; Moraes et al., 2015; Balestrin et al., 2021). However, no studies to date have focused on the molecular identification of *Eimeria* species in APPS in Brazil.

Fatoba et al. (2020) identified *E. maxima, E. tenella, E. acervulina, E. brunetti,* and *E. mitis* using a species-specific nested PCR targeting the ITS-1 gene in free-range farms in South Africa. They also reported that all their samples tested negative by *E. lata, E. nagambie,* and *E. zaria* specific protocols. In backyard flocks, 10 *Eimeria* species were identified using a capillary electrophoresis assay in Australia (Godwin & Morgan, 2015). The analyses of the ITS-2 gene sequences from our study revealed, for the first time, the presence of unidentified *Eimeria* sp., *E. nagambie,* and *E. zaria* in domestic chickens in Brazil and the detection of *E. nagambie* in South America. *Eimeria* lata, *E. nagambie,* and *E. zaria* have already been identified in several countries, including Australia (Godwin & Morgan, 2015; Morgan & Godwin, 2017), Nigeria (Jatau et al., 2016; Clark et al., 2016), India (Hinsu et al., 2018), Ghana, Tanzania, Uganda, and Zambia (Fornace et al., 2013; Clark et al., 2016), and the United States (Hauck et al., 2019; Terra et al., 2021). The only study on these three new species in South America described the identification of *E. lata* and *E. zaria* in Venezuela (Clark et al., 2016).

*Eimeria lata*-specific PCR protocol (Fornace et al., 2013) resulted in 6/79 (7.6%) positive samples. However, sequencing of PCR amplicons enabled the identification of genetic sequences most similar to *Eimeria* sp., *E. lata*, and *E. maxima* (Table 2). Another *E. lata*-specific PCR protocol (Blake et al., 2021) proved to be negative, which confirms the absence, or the presence below the PCR detection threshold, of *E. lata*.

An ITS-2 sequence amplified by *E. nagambie*-specific PCR showed 98.3% genetic similarity to *E. nagambie* (Table 2). Owing to the short number of base pairs of the PCR amplicon and the intraspecies polymorphism of the ITS-2 gene, the genetic similarity to an *E. nagambie* sequence does allow this sequence to be classified as belonging to *E. nagambie*.

NGS has recently been used to identify *Eimeria* spp. from domestic chickens, allowing the identification of all 10 species of *Eimeria* and new *Eimeria* OTUs (Hinsu et al., 2018; Hauck et al., 2019; Terra et al., 2021). Using NGS, we identified 18S rRNA sequences from *E. nagambie* and *E. zaria* in Brazil, along with the identification of nine species of *Eimeria*, including *E. necatrix*/*tenella*, and an unidentified *Eimeria* sp. A surprising result of our study was the high prevalence of *E. nagambie*. *Eimeria nagambie* was also the most common species in backyard flocks in Australia (Godwin & Morgan, 2015) and the second most prevalent species in backyard flocks in the United States (Hauck et al., 2019).

A sequence from unclassified *Eimeria* sp. identified in 34.5% of the samples presented 100% genetic similarity to the sequence of *Eimeria* sp. 2 RHa-2020 (MN073208) described by Clark et al. (2016) in commercial broiler chickens in the United States. Further studies are needed to determine if this sequence is related to new OTUs of *Eimeria* or even to novel *Eimeria* species.

The relative abundances of *Eimeria* spp. were not calculated owing to potential bias introduced by nested PCR (Hauck et al., 2019). However, the highest number of sequences obtained by NGS pertains to *E. praecox* and *E. acervulina* (Table 3), which are the species with the highest fecundity (Bumstead & Millard, 1992; Blake et al., 2021). There are no data about *E. nagambie* fecundity (Blake et al., 2021). Owing to many variables related to the fecundity of *Eimeria* spp. in domestic chickens (Williams, 1973; Williams, 2001; Jenkins et al., 2013; Xu et al., 2022), definitive inferences cannot be made by comparing the number of NGS sequences with the fecundity data of nine *Eimeria* species fecundity data available to date.

Identification of the *Eimeria* species can be presumed by analyzing the morphology and morphometry of the oocysts and macroscopic lesions, but a definitive species identification is more specific and sensitive based on the use of species-specific PCR or by genus-specific PCR followed by genetic sequencing. In this study, NGS and species-specific PCR protocols for *E. lata*, *E. nagambie*, and *E. zaria* were used for the first time in samples from Brazilian farms, which explains the lack of information on these species in studies previously published in Brazil.

This is a pioneering study of the identification of *E. nagambie*, *E. zaria*, and potential new *Eimeria* OTUs in Brazil. The finding of novel *Eimeria* species in Brazilian chicken farms provides relevant information regarding coccidiosis control, since *E. nagambie* and *E. zaria*, in addition to being pathogenic, evade immune protection conferred by *Eimeria* commercial vaccines (Venkatas & Adeleke, 2019; Blake et al., 2021).

Considering the economic relevance of coccidiosis in domestic chicken farms, further research should be performed on the prevalence of infection by *Eimeria* spp., particularly *E. lata*, *E. nagambie*, *E. zaria*, and potential new OTUs in domestic chicken farms, especially in CPPS.

## Conclusions

In conclusion, 18S rRNA-targeted NGS identified nine species of *Eimeria* from domestic chickens raised in APPS, including *E. necatrix*/*tenella*, and unidentified *Eimeria* sp. Species-specific PCR protocols targeting the ITS-2 locus followed by sequencing identified, for the first time in Brazil, *E. nagambie*, *E. zaria*, and novel sequences most similar to several *Eimeria* sp., *E. lata*, *E. maxima,* and *E. nagambie* sequences.

## References

[B001] Allgayer H, Hiller RF, Valiati VH (2021). Uma análise epistêmica para a elucidação do complexo de espécies crípticas. Conjectura: Filos Educ.

[B002] Balestrin PWG, Balestrin E, Santiani F, Biezus G, Moraes JC, Casa MS (2021). Prevalence of *Eimeria* sp. in broiler poultry houses with positive and negative pressure ventilation systems in Southern Brazil. Avian Dis.

[B003] Blake DP, Knox J, Dehaeck B, Huntington B, Rathinam T, Ravipati V (2020). Re-calculating the cost of coccidiosis in chickens. Vet Res (Faisalabad).

[B004] Blake DP, Vrba V, Xia D, Jatau ID, Spiro S, Nolan MJ (2021). Genetic and biological characterisation of three cryptic *Eimeria* operational taxonomic units that infect chickens (*Gallus gallus domesticus*). Int J Parasitol.

[B005] Bumstead N, Millard B (1992). Variation in susceptibility of inbred lines of chickens to seven species of *Eimeria.*. Parasitology.

[B006] Cantacessi C, Riddell S, Morris GM, Doran T, Woods WG, Otranto D (2008). Genetic characterization of three unique operational taxonomic units of *Eimeria* from chickens in Australia based on nuclear spacer ribosomal DNA. Vet Parasitol.

[B007] Carvalho FS, Wenceslau AA, Teixeira M, Carneiro JAM, Melo ADB, Albuquerque GR (2011). Diagnosis of *Eimeria* species using traditional and molecular methods in field studies. Vet Parasitol.

[B008] Clark EL, Macdonald SE, Thenmozhi V, Kundu K, Garg R, Kumar S (2016). Cryptic *Eimeria* genotypes are common across the southern but not northern hemisphere. Int J Parasitol.

[B009] Dong X, Kleiner M, Sharp CE, Thorson E, Li C, Liu D (2017). Fast and simple analysis of MiSeq amplicon sequencing data with MetaAmp. Front Microbiol.

[B010] Edgar RC, Haas BJ, Clemente JC, Quince C, Knight R (2011). UCHIME improves sensitivity and speed of chimera detection. Bioinformatics.

[B011] Fatoba AJ, Zishiri OT, Blake DP, Peters SO, Lebepe J, Mukaratirwa S (2020). Study on the prevalence and genetic diversity of *Eimeria* species from broilers and free-range chickens in KwaZulu-Natal province, South Africa. Onderstepoort J Vet Res.

[B012] Fornace KM, Clark EL, MacDonald SE, Namangala B, Karimuribo E, Awuni J (2013). Occurrence of *Eimeria* species parasites on small-scale commercial chicken farms in Africa and indication of economic profitability. PLoS One.

[B013] Godwin RM, Morgan JAT (2015). A molecular survey of *Eimeria* in chickens across Australia. Vet Parasitol.

[B014] Hall TA (1999). BioEdit: a user-friendly biological sequence alignment editor and analysis program for Windows 95/98/NT. Nucleic Acids Symp Ser.

[B015] Hauck R, Carrisosa M, McCrea BA, Dormitorio T, Macklin KS (2019). Evaluation of next-generation amplicon sequencing to identify *Eimeria* spp. of chickens. Avian Dis.

[B016] Hinsu AT, Thakkar JR, Koringa PG, Vrba V, Jakhesara SJ, Psifidi A (2018). Illumina next generation sequencing for the analysis of *Eimeria* populations in commercial broilers and indigenous chickens. Front Vet Sci.

[B017] Illumina (2013). 16S metagenomic sequencing library preparation.

[B018] Jatau ID, Lawal IA, Kwaga KP, Tomley FM, Blake DP, Nok AJ (2016). Three operational taxonomic units of *Eimeria* are common in Nigerian chickens and may undermine effective molecular diagnosis of coccidiosis. BMC Vet Res.

[B019] Jenkins MC, Parker C, O’Brien C, Miska K, Fetterer R (2013). Differing susceptibilities of *Eimeria acervulina, Eimeria maxima*, and *Eimeria tenella* oocysts to desiccation. J Parasitol.

[B020] Lew AE, Anderson GR, Minchin CM, Jeston PJ, Jorgensen WK (2003). Inter- and intra-strain variation and PCR detection of the internal transcribed spacer 1 (ITS-1) sequences of Australian isolates of *Eimeria* species from chickens. Vet Parasitol.

[B021] Luchese FC, Perin M, Aita RS, Mottin VD, Molento MB, Monteiro SG (2007). Prevalência de espécies de *Eimeria* em frangos de criação industrial e alternativa. Braz J Vet Res Anim Sci.

[B022] Meireles MV, Roberto LO, Riera RF (2004). Identification of *Eimeria mitis* and *Eimeria praecox* in broiler feces using polymerase chain reaction. Rev Bras Cienc Avic.

[B023] Moraes JC, França M, Sartor AA, Bellato V, Moura AB, Magalhães ML (2015). Prevalence of *Eimeria* spp. in broilers by multiplex PCR in the Southern Region of Brazil on two hundred and fifty farms. Avian Dis.

[B024] Morgan JAT, Godwin RM (2017). Mitochondrial genomes of Australian chicken *Eimeria* support the presence of ten species with low genetic diversity among strains. Vet Parasitol.

[B025] Morris GM, Woods WG, Richards DG, Gasser RB (2007). Investigating a persistent coccidiosis problem on a commercial broiler-breeder farm utilising PCR-coupled capillary electrophoresis. Parasitol Res.

[B026] Noronha PC, Carrijo DL, Santos GA, Cardozo SP (2020). Detecção e identificação de *Eimeria* sp em galinhas caipiras produzidas no município de Mineiros, Goiás. Braz J Develop.

[B027] Santana BN, Kurahara B, Nakamura AA, Camargo VS, Ferrari ED, Silva GS (2018). Detection and characterization of *Cryptosporidium* species and genotypes in three chicken production systems in Brazil using different molecular diagnosis protocols. Prev Vet Med.

[B028] Santos RFS, Kavavata GM, Almeida SM, Hisano M, Calixto LFL, Meireles MV (2003). Ocorrência de *Eimeria* sp. em frangos de corte no estado de São Paulo. Ars Vet.

[B029] Silva JT, Alvares FBV, Lima EF, Silva GM, Silva ALP, Lima BA (2022). Prevalence and diversity of *Eimeria* spp. in free-range chickens in northeastern Brazil. Front Vet Sci.

[B030] Terra AT, Costa PS, Figueiredo PC, Carvalho ECQ (2001). Freqüência de espécies do gênero *Eimeria* em frangos de corte abatidos industrialmente no município de Monte Alegre do Sul, Estado de São Paulo. Braz J Vet Parasitol.

[B031] Terra MTB, Pacheco WJ, Harrison M, McCrea BA, Hauck R (2021). A Survey of coccidia and nematodes in pastured poultry in the state of Georgia. Avian Dis.

[B032] The Galaxy Community (2022). The Galaxy platform for accessible, reproducible and collaborative biomedical analyses: 2022 update. Nucleic Acids Res.

[B033] Venkatas J, Adeleke MA (2019). Emerging threat of *Eimeria* operational taxonomic units (OTUs) on poultry production. Parasitology.

[B034] Vrba V, Poplstein M, Pakandl M (2011). The discovery of the two types of small subunit ribosomal RNA gene in *Eimeria mitis* contests the existence of *E. mivati* as an independent species. Vet Parasitol.

[B035] Williams RB (1999). A compartmentalised model for the estimation of the cost of coccidiosis to the world’s chicken production industry. Int J Parasitol.

[B036] Williams RB (2001). Quantification of the crowding effect during infections with the seven *Eimeria* species of the domesticated fowl: its importance for experimental designs and the production of oocyst stocks. Int J Parasitol.

[B037] Williams RB (1973). The effect of *Eimeria acervulina* on the reproductive potentials of four other species of chicken coccidia during concurrent infections. Br Vet J.

[B038] Xu L, Xiang Q, Li M, Sun X, Lu M, Yan R (2022). Pathogenic effects of single or mixed infections of *Eimeria mitis, Eimeria necatrix*, and *Eimeria tenella* in chickens. Vet Sci.

